# Association between preoperative serum zinc level and prognosis in patients with advanced esophageal cancer in the neoadjuvant treatment era

**DOI:** 10.1002/ags3.12781

**Published:** 2024-03-06

**Authors:** Yuto Kubo, Shota Igaue, Daichi Utsunomiya, Kentaro Kubo, Daisuke Kurita, Koshiro Ishiyama, Junya Oguma, Hiroyuki Daiko

**Affiliations:** ^1^ Department of Esophageal Surgery National Cancer Center Hospital Tokyo Japan

**Keywords:** esophageal cancer, essential trace element, neoadjuvant chemotherapy, recurrence, zinc

## Abstract

**Background:**

Zinc (Zn), an essential trace element, has an adverse influence on the prognosis of several cancers. However, the association between the preoperative serum Zn level and outcomes in patients with advanced esophageal cancer in the current neoadjuvant treatment era remains unclear.

**Methods:**

This study involved 185 patients with esophageal cancer who underwent R0 surgery after neoadjuvant chemotherapy from August 2017 to February 2021. We retrospectively investigated the relationship between the preoperative serum Zn level and the patients' outcomes.

**Results:**

The patients were divided into a low Zn group (<64 μg/dL) and a high Zn group (≤64 μg/dL) according to the mean preoperative serum Zn level. Low Zn had significantly worse overall survival (OS) (2‐year OS rate: 76.2% vs. 83.3% in low vs. high Zn; *p* = 0.044). A low Zn in pathological non‐responders (Grade ≤ 1a) was significantly associated with a shorter 2‐year recurrence‐free survival (RFS) rate (39.6% vs. 64.1% in low vs. high Zn; *p* = 0.032). The multivariate analysis identified low BMI and Zn level among preoperative nutritional status indices as an independent risk factor for worse RFS in non‐responders. Compared with responders, pathological non‐responders comprised significantly more males and a performance status of ≥1, and there was no difference in Zn level according to pathological response.

**Conclusion:**

A preoperative low Zn level had a negative impact on early recurrence in esophageal cancer patients who underwent neoadjuvant chemotherapy. This suggests the need to administer Zn supplementation to patients with esophageal cancer who have preoperative Zn deficiency.

## Graphical abstract

The preoperative low serum Zn level was significantly associated with poor prognosis. A low Zn level as independent factors associated with worse recurrence‐free survival in non‐responders after R0 surgery.

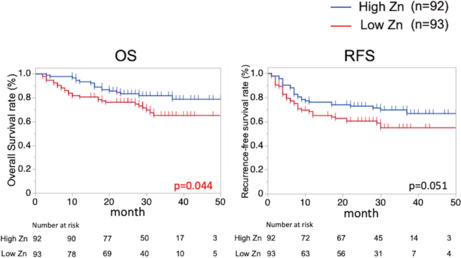

## 1 INTRODUCTION

Esophageal cancer is the eighth most common cancer worldwide and has the sixth worst prognosis because of its aggressiveness and low survival rate. The morbidity of esophageal cancer has continued to increase during the past three decades,^1^, ^2^ In East Asian countries, including Japan, neoadjuvant chemotherapy (NAC) has been a standard treatment to control microscopic metastases, downstage tumors, and improve resectability.^3^

The perioperative nutritional status is associated with the short‐term outcomes and prognosis in esophageal cancer after open and minimally invasive esophagectomy.^2^, ^4^, ^5^ Additionally, a poor preoperative nutritional status adversely affects postoperative complications as well as overall survival after esophageal cancer surgery.^5^, ^6^ Thus, it is necessary to evaluate the nutritional status in these patients because of its influence on patients' quality of life and prognosis.

Zinc (Zn) is an essential trace element that has important roles in immune system function.^7^ Recent in vitro studies have shown that the Zn level is associated with chemoresistance.^8^, ^9^ Additionally, the serum Zn level has an adverse influence on the prognosis of colon cancer and liver cancer.^10^, ^11^ However, the association between the preoperative serum Zn level and outcomes in esophageal cancer patients who have undergone NAC remains unclear.

Hence, the present study focused on patients with esophageal cancer who underwent NAC and whose preoperative serum Zn level was associated with their long‐term outcomes. The aim of this retrospective study was to validate whether the preoperative serum Zn level can predict the prognosis for patients with esophageal cancer after NAC.

## 2 MATERIALS AND METHODS

### 2.1 Patients

From August 2017 to February 2021, 332 patients with thoracic esophageal cancer underwent esophagectomy at National Cancer Center Hospital. This study evaluated the preoperative blood sample, including serum Zn level, at 2–3 days before surgery. Of these 332 patients, we excluded 26 who underwent salvage surgery, 87 who did not undergo NAC because of early esophageal cancer, five who underwent neoadjuvant chemoradiation therapy, seven in whom the preoperative serum Zn level, and 22 who performed R1 and R2 surgery could not be evaluated. Thus, 185 patients were included in the present study. Before and after neoadjuvant treatment, the patients were staged by computed tomography (CT) and endoscopy. The clinicopathological findings were classified according to the Union for International Cancer Control (UICC) TNM classification, eighth edition.^12^ The clinical responses were classified based on the World Health Organization response criteria and the criteria of the Japanese Society for Esophageal Disease using CT and endoscopy.^13^, ^14^ Additionally, the patients' comorbidities were quantified by the Charlson Comorbidity Index, which is a commonly used score that quantifies multiple comorbidities.^15^

### 2.2 Neoadjuvant therapy and surgical procedure

NAC followed by surgery at our hospital was performed for patients with cStage I (excluding T1N0), II, III, or IV esophageal cancer without distant organ metastasis. The NAC regimen consisted of three cycles of 5‐fluorouracil and cisplatin plus docetaxel (i.e., DCF) or two cycles of 5‐fluorouracil and cisplatin (i.e., CF) as described in the JCOG1109 randomized controlled phase 3 trial^16^ or another regimen including chemotherapy plus immunotherapy.^17^ Each course of chemotherapy was administered with a 2‐ to 3‐week rest period.

The surgical procedure for patients with thoracic esophageal cancer was subtotal esophagectomy. Our hospital generally uses minimally invasive esophagectomy methods, and the selection of thoracoscopic surgery, robotic surgery, or mediastinoscopy was determined according to the patients' general condition as indicated by parameters such as age, comorbidities, and cStage.^18^, ^19^ Patients who required lymph node dissection generally underwent three‐field lymph node dissection regardless of the esophageal cancer location.

### 2.3 Postoperative follow‐up

Regard of the pathological response, the percentage of viable residual tumor cells of esophageal cancer was classified into five categories as follows: grade 3; no residual tumor cells, grade 2; <1/3 residual tumor cells, grade 1b; 1/3 to 2/3 residual tumor cells, grade 1a; >2/3 residual tumor cells and grade 0; no response to neoadjuvant chemotherapy.^14^ Postoperative complications were defined as Clavien–Dindo grade ≥ II complications.^20^ Postoperative follow‐up of all patients was performed at intervals of 3 to 4 months during the first 2 years and every 6 months for another 3 years. CT scans and tumor markers were evaluated every 3 to 4 months during the first 2 years and every 6 months for another 3 years until 5 years after the esophagectomy. Upper gastrointestinal endoscopy was performed annually to check for recurrence at the anastomotic site and in the gastric conduit. When the CT results indicated recurrence, further investigations were performed by more selective methods such as [^18^F]fluorodeoxyglucose positron emission tomography, bone scintigraphy, and magnetic resonance imaging. The observation period for all patients was from August 2017 to February 2023.

### 2.4 Nutritional status

This study employed several nutritional indices that can be used to objectively evaluate the nutritional status before surgery: the Zn level, albumin (Alb) level, prognostic nutritional index (PNI), C‐reactive protein/Alb ratio, and Controlling Nutritional Status (CONUT) score. The PNI is calculated using the serum Alb level and the total lymphocyte count (PNI = 10 × Alb + 0.005 × lymphocyte count).^21^ The CONUT score is calculated using the Alb level, total lymphocyte count, and total cholesterol level and is evaluated as follows: score of 0–1, normal; score of 2–4, mild; score of 5–8, moderate; and score of ≥9, severe.^22^

### 2.5 Statistical analysis

The Mann–Whitney *U* test, *χ* ^2^ test, or Student's *t* test was used to compare patient characteristics. The overall survival (OS) and recurrence‐free survival (RFS) rates were calculated from the first day of surgery, validated by the Kaplan–Meier method, and compared with the log‐rank test on an intent‐to‐treat basis; the corresponding hazard ratios were calculated with the 95% confidence intervals. Cox proportional hazards regression models were used to identify variables significantly associated with the prognosis. Continuous variables are expressed as mean ± standard deviation unless otherwise stated. Statistical significance was considered at a *p* value of <0.05. All analyses were performed using JMP® 14 (SAS Institute Inc., Cary, NC, USA).

## 3 RESULTS

### 3.1 Preoperative serum Zn level

The mean preoperative serum Zn level was 64 μg/dL; therefore, the patients were divided into two groups: those with a preoperative serum Zn level of <64 μg/dL (low Zn group) and those with a preoperative serum Zn level of ≥64 μg/dL. Of the 185 patients included in our study, the low Zn group comprised 93 patients and the high Zn group comprised 92 patients (Figure S1).

### 3.2 Patient characteristics

The two groups had no significant differences in age, preoperative body mass index (BMI), performance status (PS), location of esophageal cancer, histological type, Charlson Comorbidity Index, or C‐reactive protein/Alb ratio. However, the numbers of male patients and patients with advanced esophageal cancer (cStage III) were significantly higher in the low Zn group than in the high Zn group. Additionally, hemoglobin (Hb) level, nutritional status indices, including the Alb level and PNI, were significantly lower in the low Zn group than in the high Zn group (Table 1).

**TABLE 1:** Baseline patient characteristics according to preoperative Zn.

	All patients	Low Zn	High Zn	*p* value
No. of patient (*n*, %)	185	93 (50.3%)	92 (49.7%)	
Age	64.1 ± 9.3	64.9 ± 9.3	63.9 ± 9.9	0.453
Sex				
Male	149	83 (89.3%)	66 (71.7%)	0.003
Female	36	10 (10.7%)	26 (28.3%)	
BMI	22.1 ± 3.8	22.2 ± 4.0	22.4 ± 3.7	0.481
PS				
0	165	81 (87.1%)	84 (91.3%)	0.088
1	18	12 (12.9%)	6 (6.5%)	
2	2	0	2 (1.9%)	
Location				
Ut	36	14 (15.1%)	22 (23.9%)	0.205
Mt	90	45 (48.4%)	45 (48.9%)	
Lt	59	34 (36.6%)	25 (25.2%)	
cStage (TNM)				
I	23	6 (6.5%)	17 (18.5%)	0.047
II	27	12 (12.9%)	15 (16.3%)	
III	105	60 (64.5%)	45 (48.9%)	
IV	30	15 (16.1%)	15 (16.3%)	
Histological type				
SCC	164	80 (86.0%)	84 (91.3%)	0.188
Adenocarcinoma	19	11 (11.8%)	8 (8.7%)	
Other	2	2 (2.2%)	0	
CCI^a^				
Score 0	94	44 (47.3%)	50 (54.4%)	0.338
Score ≥ 1	91	49 (52.7%)	42 (45.6%)	
Hb^a^ (g/dL)	11.8 ± 1.4	11.5 ± 1.4	12.2 ± 1.4	<0.001
Alb^a^ (g/dL)	3.9 ± 0.4	3.8 ± 0.4	4.1 ± 0.4	<0.001
PNI^a^	46.4 ± 5.1	45.4 ± 4.6	48.1 ± 4.9	<0.001
CRP/albumin ratio^a^	0.13 ± 0.28	0.17 ± 0.30	0.08 ± 0.19	0.091
CONUT score				
Score ≤ 4	179	88 (94.6%)	91 (98.9%)	0.086
Score ≥ 5	6	5 (5.4%)	1 (1.1%)	

*Note*: Data are the mean ± SD.

Abbreviations: Alb, albumin; BMI, body mass index; CCI, Charlson Comorbidity Index; CONUT, Controlling Nutritional Status; CRP, C‐reactive protein; cStage: clinical stage; Hb, hemoglobin; PNI, Prognostic Nutritional Index; PS, performance status.

^a^ Preoperative data.

### 3.3 Treatment factors

Table 2 shows the details of treatments such as NAC and surgery. The two groups showed no significant differences in the clinical treatment effect; surgical approach; residual tumor; two‐stage esophagectomy; reconstruction route; reconstruction organ; postoperative complications such as pneumonia, nerve palsy, and chylothorax; pStage; and pathological treatment effect. However, significantly more patients in the low than high Zn group received DCF as their NAC regimen. The incidence rate of postoperative anastomotic leakage was significantly lower in the low than high Zn group.

**TABLE 2:** Details of treatment in the low Zn group and high Zn group.

	All patients	Low Zn	High Zn	*p* value
No. of patients	185	93 (50.3%)	92 (49.7%)	
Neoadjuvant chemotherapy				
DCF	101	59 (63.4%)	42 (45.6%)	0.010
FP	61	21 (22.6%)	40 (43.5%)	
other	23	13 (14.0%)	10 (10.9%)	
Clinical treatment effect				
CR	3	0	3 (3.3%)	0.162
PR	134	66 (71.0%)	68 (73.9%)	
SD	35	19 (20.4%)	16 (17.4%)	
PD	13	8 (8.6%)	5 (5.4%)	
Surgical approach				
Thoracoscopy	156	82 (88.2%)	74 (80.4%)	0.214
Mediastinoscopy	1	0	1 (1.1%)	
Robot	28	11 (11.8%)	17 (18.5%)	
Two‐stage esophagectomy	3	1 (1.1%)	2 (2.2%)	0.551
Reconstruction route				
Antethoracic	1	1 (1.1%)	0	0.465
Retrosternal	177	89 (95.7%)	88 (95.7%)	
Posterior mediastinal	7	3 (3.2%)	4 (4.4%)	
Reconstruction organ				
Stomach tube	175	88 (94.6%)	87 (94.6%)	0.986
Other	10	5 (5.4%)	5 (5.4%)	
Postoperative complication^a^				
Pneumonia	47	24 (25.8%)	23 (25.0%)	0.900
Anastomotic leakage	19	4 (4.3%)	15 (16.3%)	0.006
Recurrent nerve palsy	21	12 (12.9%)	9 (9.8%)	0.503
Chylothorax	23	15 (16.1%)	8 (8.7%)	0.123
pStage (TNM)				
0	20	10 (10.8%)	10 (10.8%)	0.423
I	35	14 (15.1%)	21 (22.8%)	
II	52	24 (25.8%)	28 (30.4%)	
III	64	36 (38.7%)	28 (30.4%)	
IV	14	9 (9.7%)	5 (5.4%)	
Pathological treatment effect				
Responder (Grade ≤ 1b)	105	52 (55.9%)	53 (57.6%)	0.816
Non‐responder (Grade ≤ 1a)	80	41 (44.1%)	39 (42.4%)	

Abbreviations: CR, complete response; PD, progressive disease; PR, partial response; pStage, pathological stage; SD, stable disease.

^a^ Clavien–Dindo classification Grade ≥ II.

### 3.4 OS and RFS

**FIGURE 1:**
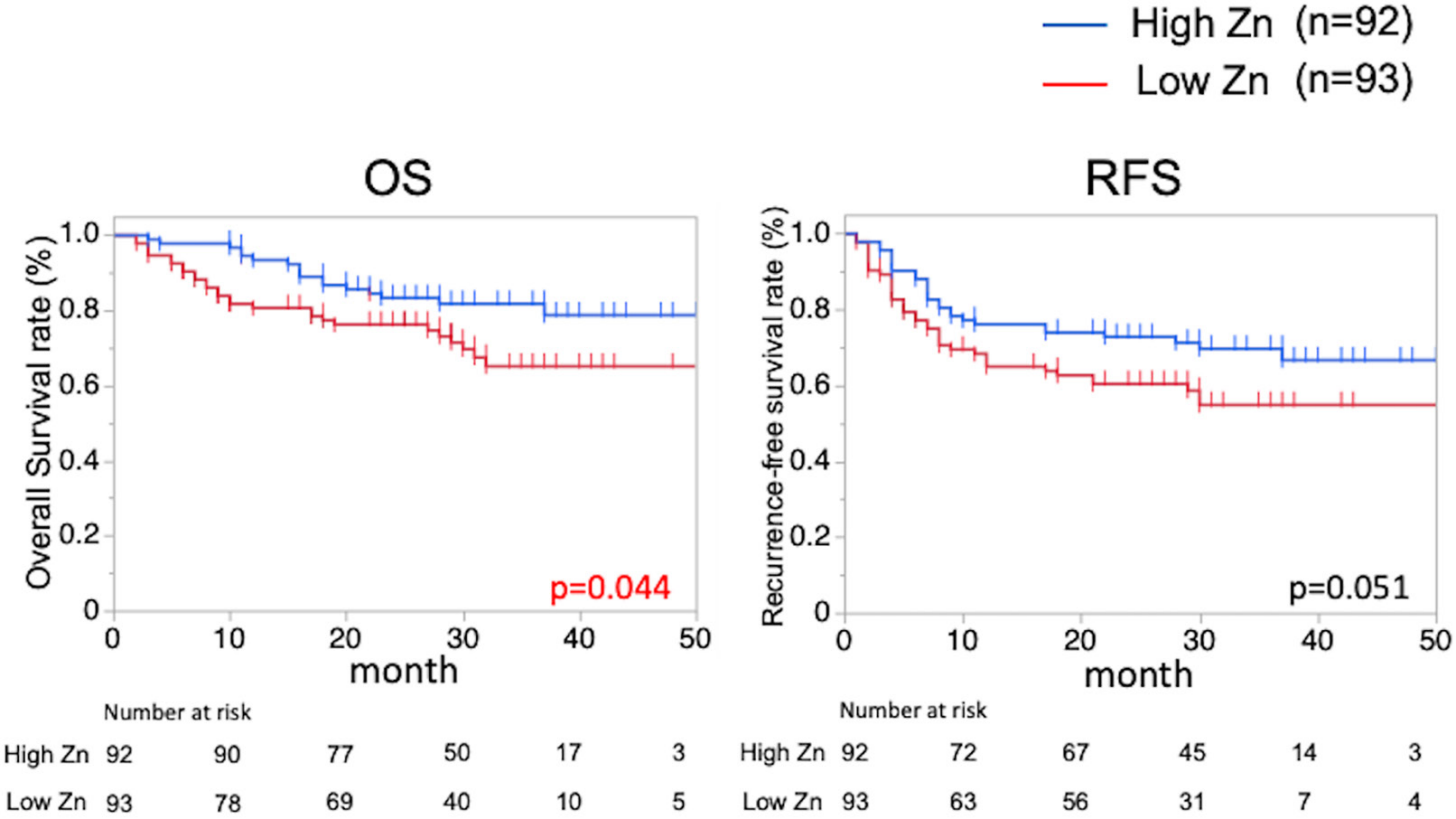
OS and RFS between the low Zn group and the high Zn group in R0 surgery. OS, overall survival; RFS, recurrence‐free survival; Zn, zinc.

Figure 1 shows the Kaplan–Meier estimates of cumulative OS and RFS for patients with R0 surgery in the two groups. The median duration of follow‐up after R0 surgery was no significant difference in low and high Zn group (26 months vs. 28 months, *p* = 0.251). Low Zn was significantly associated with shorter OS (2‐year OS rate in low vs. high Zn group: 76.2% vs. 83.3%, respectively; *p* = 0.044). Additionally, RFS tended to be shorter in the low Zn group than in high Zn group (2‐year RFS rate in low vs. high Zn group: 60.3% vs. 72.7%, respectively; *p* = 0.051). However, multivariate analysis indicated that preoperative Zn level was not an independent factor for OS and RFS after R0 surgery. Also, no significant differences in metastatic lesions after surgery or treatment after recurrence were found between the two groups after R0 surgery (Table S1).

**FIGURE 2:**
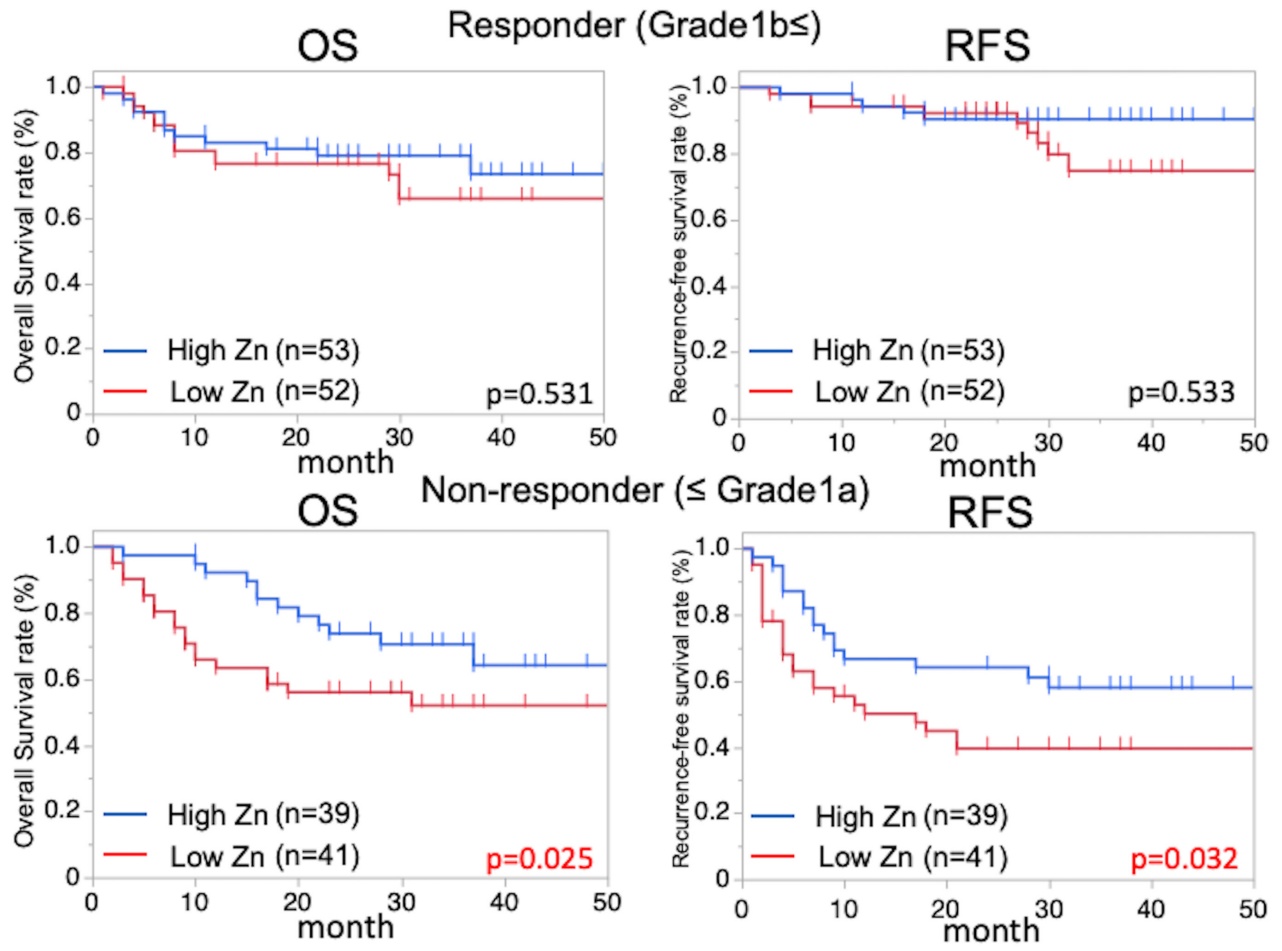
OS and RFS between the low Zn group and the high Zn group in pathological responders and non‐responders after R0 surgery. OS, overall survival; RFS, recurrence‐free survival; Zn, zinc.

Also, there was no significant difference in OS and RFS between low and high Zn groups in clinical responders (i.e., complete response and partial response) and non‐responders (i.e., stable disease and progressive disease), respectively (Figure S2). On the other hand, according to the pathological treatment effect (i.e., responders [Grade ≥ 1b] and non‐responders [Grade ≤ 1a]), a low Zn level in non‐responders was significantly associated with shorter OS and RFS (2‐year OS rate in low vs. high Zn group: 56.0% vs. 73.8%, respectively; *p* = 0.025, 2‐year RFS rate in low vs. high Zn group: 39.6% vs. 64.1%, respectively; *p* = 0.032), although there was no difference in OS and RFS of responders between the two groups (Figure 2). Multivariate analyses identified only a low Zn level and preoperative BMI as independent factors associated with worse RFS in non‐responders after R0 surgery (Table 3). Also, responders and non‐responders had similar follow‐up median duration in the two groups (responder: low Zn group vs. high Zn group; 26 months vs. 27 months, *p* = 0.345, non‐ responder: low Zn group vs. high Zn group; 24 months vs. 25 months, *p* = 0.576).

**TABLE 3:** Uni‐/multivariate analyses of progression‐free survival in esophageal cancer patients with non‐responder after R0 surgery.

Variables	Univariate analysis			Multivariate analysis		
	OR	95% CI	*p* Value	OR	95% CI	*p* Value
Age ≥ 65 (vs. <65)	1.269	0.682–2.360	0.452			
Sex male (vs. female)	1.888	0.582–6.127	0.290			
PS 1 (vs. class 0)	1.474	0.577–3.763	0.417			
CCI ≥ score 1 (vs. score 0)	1.374	0.738–2.558	0.316			
BMI^a^ < 22 (vs. ≥22)	2.142	1.133–4.049	0.019	2.386	1.215–4.684	0.012
Location Ut, Mt (vs. Lt)	1.126	0.593–2.136	0.717			
cStage III, IV (vs. cStage I, II)	1.619	0.746–3.516	0.223			
Hb^a^ < 11.0 g/dL (vs. ≥11.0 g/dL)	1.628	0.827–2.702	0.181			
Alb^a^ < 3.5 g/dL (vs. ≥3.5 g/dL)	1.906	0.950–3.827	0.070	1.713	0.628–4.671	0.293
PNI^a^ < 47 (vs. ≥47)	1.436	0.770–2.680	0.256			
CONUT score ≤1 (vs. ≥2)	1.637	0.502–5.340	0.414			
Low Zn group (vs. High Zn group)	1.965	1.024–3.772	0.042	2.025	1.043–3.931	0.037

Abbreviations: Alb, albumin; BMI, body mass index; CCI, Charlson Comorbidity Index; CI, confidence interval; CONUT, Controlling Nutritional Status; Hb: hemoglobin; OR, odds ratio; PNI: Prognostic Nutritional Index; PS, performance status.

^a^ Preoperative data.

### 3.5 Risk factors for pathological non‐responder status

The characteristics of pathological non‐responders and responders are summarized in Table 4. The age, preoperative BMI, location of esophageal cancer, cT, cN, cM, cStage, and histological type were similar between non‐responders and responders. On the other hand, pathological non‐responders comprised significantly more male patients and patients with a PS of ≥1 (male: 88.8% vs. female: 11.2%, *p* = 0.012 and PS of ≥1: 17.6% vs. 5.7%, *p* = 0.031). There was no significant difference in CCI, Hb level, Alb level, PNI, CONUT score, and Zn level in non‐responder and responder.

**TABLE 4:** Patient characteristics according to the status of pathological response.

	Non‐responder	Responder	*p* value
No. of patient (*n*, %)	80 (43.2%)	105 (56.7%)	
Age	65.5 ± 8.7	63.6 ± 10.2	0.414
Sex			
Male	71 (88.8%)	78 (74.3%)	0.012
Female	9 (11.2%)	27 (25.7%)	
BMI^a^	22.0 ± 3.6	22.5 ± 4.0	0.533
PS			
0	66 (82.5%)	99 (94.3%)	0.031
1	13 (16.3%)	5 (4.8%)	
2	1 (1.3%)	1 (0.9%)	
Location			
Ut	13 (16.3%)	23 (21.9%)	0.443
Mt	38 (47.5%)	52 (49.5%)	
Lt	29 (36.2%)	30 (28.6%)	
cT			
1, 2	17 (21.3%)	30 (28.6%)	0.254
3, 4	63 (78.7%)	75 (71.4%)	
cN			
0, 1	47 (58.8%)	70 (66.7%)	0.269
2, 3	33 (41.2%)	35 (33.3%)	
cM			
0	70 (87.5%)	93 (88.6%)	0.824
1	10 (12.5%)	12 (11.4%)	
cStage (TNM)			
I, II	20 (25.0%)	30 (28.6%)	0.587
III, IV	60 (75.0%)	75 (71.4%)	
Histological type			
SCC	67 (83.8%)	97 (92.4%)	0.177
Adenocarcinoma	12 (15.0%)	7 (6.7%)	
Other	1 (1.3%)	1 (0.9%)	
CCI^a^			
Score 0	44 (55.0%)	50 (47.6%)	0.320
Score ≥ 1	36 (45.0%)	55 (52.4%)	
Hb^a^ (g/dL)	11.9 ± 1.4	11.8 ± 1.5	0.368
Alb^a^ (g/dL)	3.9 ± 0.4	3.9 ± 0.4	0.459
PNI^a^	46.4 ± 5.2	47.0 ± 4.7	0.505
CONUT score^a^			
Score ≤ 4	76 (95.0%)	103 (98.1%)	0.240
Score ≥ 5	4 (5.0%)	2 (1.9%)	
Zn^a^ (μg/dL)	65.0 ± 12.2	62.5 ± 10.4	0.581

*Note*: Data are the mean ± SD.

Abbreviations: Alb, albumin; BMI, body mass index; CCI, Charlson Comorbidity Index; CONUT, Controlling Nutritional Status; cStage, clinical Stage; Hb, hemoglobin; PNI, Prognostic Nutritional Index; PS, performance status; Zn, Zinc.

^a^ Preoperative data.

## 4 DISCUSSION

This study showed that the preoperative serum Zn level had a negative association with early recurrence of esophageal cancer. Especially in pathological non‐responders, the preoperative serum Zn level had a worse impact on early recurrence. Moreover, among the preoperative nutritional status indices, only the Zn level was an independent factor for recurrence in pathological non‐responders. Previous studies have demonstrated the association between the Zn level and prognosis in several types of cancer; however, the influence of the serum Zn level on the prognosis in esophageal cancer patients who undergo NAC remains unclear. The present study showed that a low serum Zn level before surgery had a worse influence on RFS. Therefore, we consider that the preoperative serum Zn level may serve as a predictive marker for early recurrence after esophageal cancer surgery.

Zn deficiency caused by malabsorption syndrome and malnutrition is associated with several types of cancer. Wu et al.^11^ showed that patients who had Stage I to III colon cancer with a Zn level of <85.6 μg/dL had a worse prognosis as indicated by a significantly higher incidence of distant metastasis. Additionally, Imai et al.^10^ reported that patients who had undergone initial hepatectomy for treatment of hepatocellular carcinoma and had a low preoperative serum Zn level of ≤65 μg/dL had significantly lower disease‐free survival and OS rates than those with a high Zn level of >65 μg/dL, and the serum Zn level was an independent prognostic factor for OS. We found that a preoperative serum Zn level of less than the mean value (<64 mg/dL) was associated with poor RFS. These results suggest the need to evaluate the serum Zn level before surgery in patients with gastrointestinal cancer.

Several in vitro and in vivo studies have focused on the relationship between Zn and esophageal cancer. Zn deficiency induced single‐ and double‐strand DNA breaks, increased oxidative stress or inflammation, and impaired immune function in esophageal cancer.^23^, ^24^ In another study, Zn loss promoted the proliferation of esophageal squamous cells and decreased the growth rate in mice.^25^ These findings indicate that the reason why a low serum Zn level is associated with greater recurrence of esophageal cancer may be due to the increased malignancy and metastasis potential caused by Zn deficiency. Also, this study indicated that DCF was significantly superior to CF in RFS by univariate analysis (HR: 0.696, 95% CI: 0.356–0.921, *p* = 0.022) as well as JCOG 1109 study. However, low Zn group with significantly more DCF had poor OS and RFS compared with high Zn group. Based on this result, we consider that low serum Zn level may induce more chemoresistance and invasion capacity by triplet regimen.

The necessity for Zn preparations has been reported in patients with gastrointestinal cancer. One study showed that oral administration of Zn increased the serum Zn level and inhibited the development of hepatocellular carcinoma in patients with chronic liver disease.^26^ Another study showed that Zn supplementation combined with chemoradiation therapy effectively reduced local tumor recurrence and improved OS of patients with advanced nasopharyngeal carcinoma.^27^ However, the influence of Zn supplementation on patients with cancer who have undergone NAC remain unclear. One in vitro study showed that Zn administration prevented cell proliferation in esophageal squamous cell cancer patients,^28^ and another study showed that the administration of Zn combined with chemotherapy inhibited epithelial–mesenchymal transition, which is related to chemoresistance and improved chemotherapy sensitivity in patients with prostate cancer.^8^ Based on our results, we consider that pathological non‐responder patients associated with poor PS in this study. Hence, Zn supplementation in non‐responders after NAC (i.e., patients with a poor PS) may prevent recurrence of esophageal cancer. We conduct interventions in the form of rehabilitation and supplementation for patients with advanced esophageal cancer in the current neoadjuvant treatment era, and we are considering a prospective intervention study for patients with esophageal cancer undergoing esophagectomy in which Zn supplements are consumed for several months before surgery. The goal of such a study would be to determine whether long‐term Zn supplementation support can improve patients' nutritional status and prognosis.

This study had several limitations. First, this was a retrospective study in a single hospital. Second, we did not evaluate the serum Zn level before NAC and after surgery. We are prospectively investigating pretreatment and postoperative serum Zn level for esophageal cancer. Third, we did not investigate various nutritional markers such as oral or total intake and skeletal muscle mass.

In conclusion, we showed that a preoperative low Zn level had a negative impact on early recurrence in patients with esophageal cancer who underwent NAC and esophagectomy. We consider that Zn supplementation may be necessary for patients with preoperative Zn deficiency.

## AUTHOR CONTRIBUTIONS

Yuto Kubo designed the study and drafted paper. Hiroyuki Daiko conceptualized the project, supervised the experimental design, and interpreted the results. All authors revised the paper and approved the final version.

## FUNDING INFORMATION

None.

## CONFLICT OF INTEREST STATEMENT

There are no conflicts of interest.

## ETHICS STATEMENT

Approval of the research protocol: This study was approved by the appropriate institutional review boards of National Cancer Center Hospital (approval number: 2020‐287) and was conducted in accordance with the Declaration of Helsinki.

## Supporting information


**TABLE S1.** Clinical course according to preoperative serum Zn in patients after R0 surgery.

**FIGURE S1.** Division into the low Zn group and the high Zn group in this study.

**FIGURE S2.** OS and RFS in the low Zn group and the high Zn group of clinical responders and non‐responders.
