# Supplementary material for: Association between preoperative serum zinc level and prognosis in patients with advanced esophageal cancer in the neoadjuvant treatment era
Source: Ann Gastroenterol Surg. 2024 Mar 6;8(4):595–603. doi: 10.1002/ags3.12781 (PMC11216781; doi:10.1002/ags3.12781)
Supplement: Supplementary file 1 — TABLE S1. Clinical course according to preoperative serum Zn in patients after R0 surgery. [file AGS3-8-595-s001.docx]

Supplemental Table 1

Clinical course according to preoperative serum Zn in patients after R0 surgery

|  | Low Zn | High Zn | p value |
| --- | --- | --- | --- |
| No. of patients | 93 (50.3%) | 92 (49.7%) |  |
| No. of patients who died | 29 (31.2%) | 17 (18.5%) | 0.045 |
| No. of patients with disease recurrence | 38 (40.9%) | 28 (30.4%) | 0.113 |
| Recurrence-free survival time (months) | 21.2 ± 14.4 | 26.2 ± 14.1 | 0.010 |
| Metastatic lesion |  |  |  |
| local | 1 (2.6%) | 3 (10.7%) | 0.172 |
| lymph nodes | 21 (55.3%) | 16 (57.1%) | 0.879 |
| lung | 8 (21.1%) | 5 (17.9%) | 0.746 |
| liver | 9 (23.7%) | 6 (21.4%) | 0.829 |
| bone | 4 (10.5%) | 2 (7.1%) | 0.633 |
| dissemination | 2 (5.3%) | 1 (3.6%) | 0.742 |
| other | 4 (10.5%) | 5 (17.9%) | 0.349 |
| Treatment after recurrence |  |  |  |
| surgery | 3 (7.9%) | 1 (3.6%) | 0.684 |
| chemotherapy/radiation therapy/  chemoradiation therapy | 30 (79.0%) | 22 (78.6%) |  |
| none | 5 (13.2%) | 5 (17.9%) |  |

Data are the mean ± SD, * include chemotherapy, radiation therapy or radiation chemotherapy
