# Supplementary figures and images for: Association between preoperative serum zinc level and prognosis in patients with advanced esophageal cancer in the neoadjuvant treatment era
Source: Ann Gastroenterol Surg. 2024 Mar 6;8(4):595–603. doi: 10.1002/ags3.12781 (PMC11216781; doi:10.1002/ags3.12781)

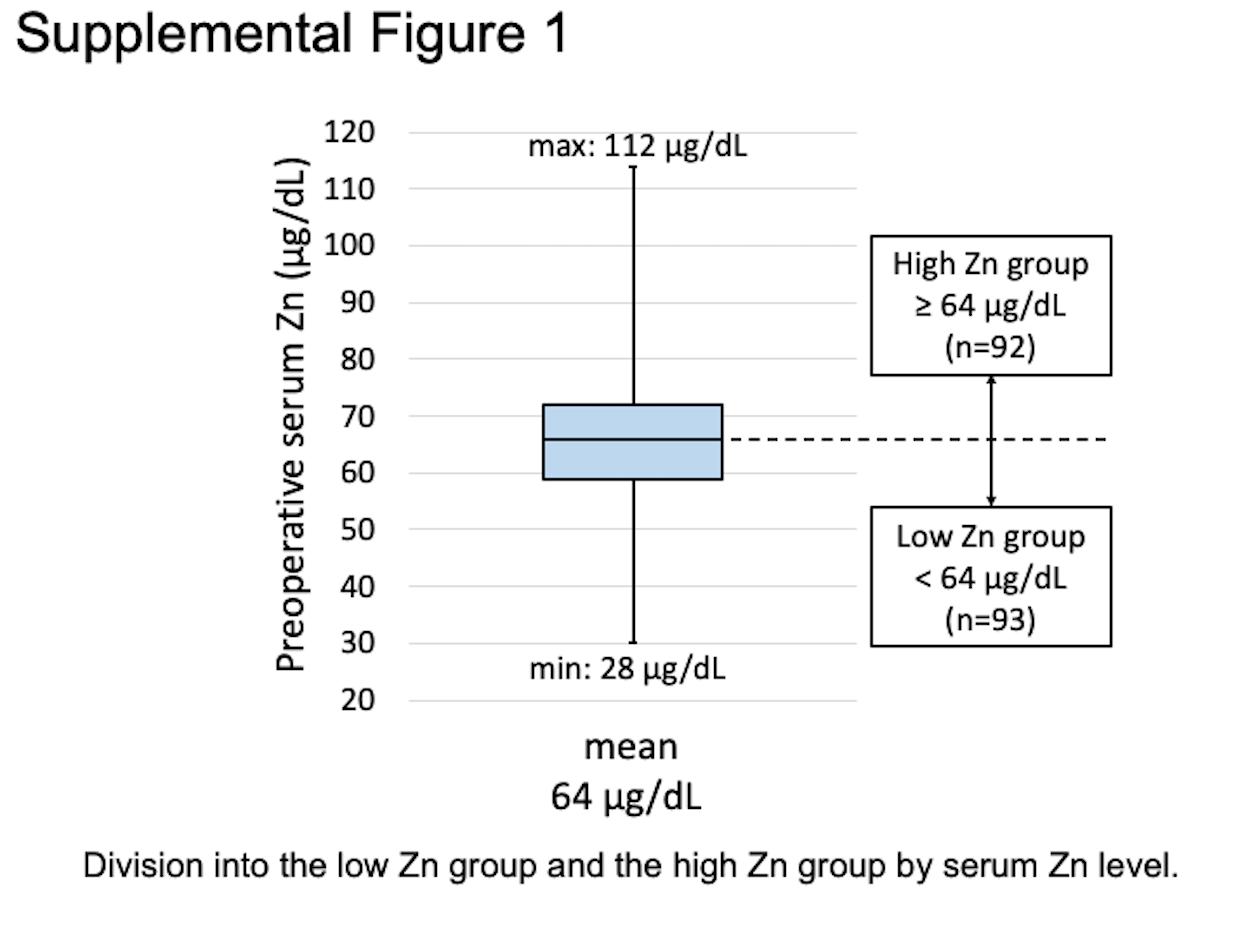

Supplement: Supplementary file 2 — FIGURE S1. Division into the low Zn group and the high Zn group in this study. [file AGS3-8-595-s002.tiff]

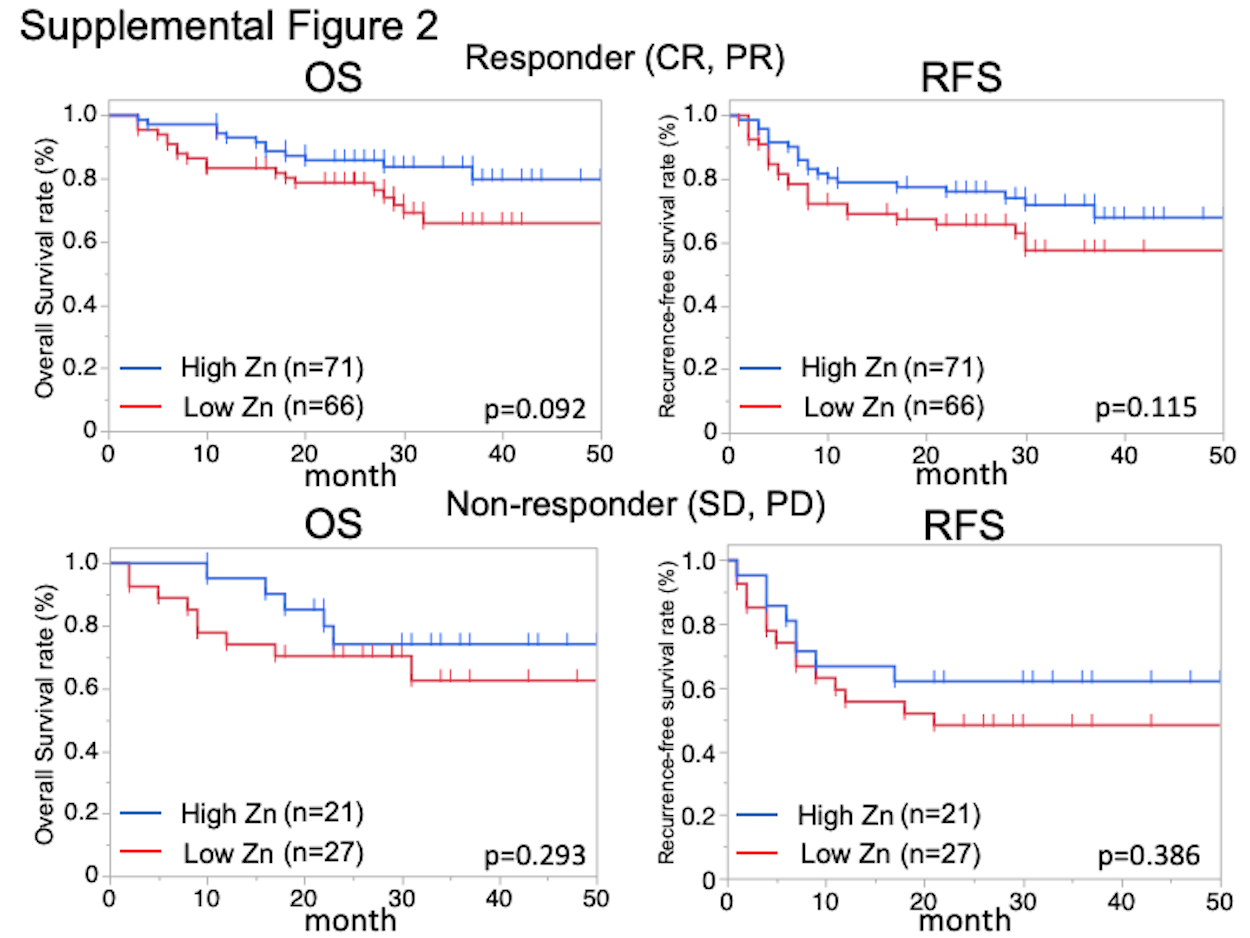

Supplement: Supplementary file 3 — FIGURE S2. OS and RFS in the low Zn group and the high Zn group of clinical responders and non‐responders. [file AGS3-8-595-s003.tiff]
